# The Emperor's New Clothes: PDE5 and the Heart

**DOI:** 10.1371/journal.pone.0118664

**Published:** 2015-03-06

**Authors:** Chantal V. Degen, Kalkidan Bishu, Rosita Zakeri, Ozgur Ogut, Margaret M. Redfield, Frank V. Brozovich

**Affiliations:** Mayo Medical School, Department of Cardiovascular Diseases, Rochester, MN, 55905, United States of America; Virginia Commonwealth University Medical Center, UNITED STATES

## Abstract

Phosphodiesterase-5 (PDE5) is highly expressed in the pulmonary vasculature, but its expression in the myocardium is controversial. Cyclic guanosine monophosphate (cGMP) activates protein kinase G (PKG), which has been hypothesized to blunt cardiac hypertrophy and negative remodeling in heart failure. Although PDE5 has been suggested to play a significant role in the breakdown of cGMP in cardiomyocytes and hence PKG regulation in the myocardium, the RELAX trial, which tested effect of PDE5 inhibition on exercise capacity in patients with heart failure with preserved ejection fraction (HFpEF) failed to show a beneficial effect. These results highlight the controversy regarding the role and expression of PDE5 in the healthy and failing heart. This study used one- and two-dimensional electrophoresis and Western blotting to examine PDE5 expression in mouse (before and after trans-aortic constriction), dog (control and HFpEF) as well as human (healthy and failing) heart. We were unable to detect PDE5 in any cardiac tissue lysate, whereas PDE5 was present in the murine and bovine lung samples used as positive controls. These results indicate that if PDE5 is expressed in cardiac tissue, it is present in very low quantities, as PDE5 was not detected in either humans or any model of heart failure examined. Therefore in cardiac muscle, it is unlikely that PDE5 is involved the regulation of cGMP-PKG signaling, and hence PDE5 does not represent a suitable drug target for the treatment of cardiac hypertrophy. These results highlight the importance of rigorous investigation prior to clinical trial design.

## Introduction

There are over five million Americans with heart failure (HF), and a significant proportion have refractory, end-stage HF unresponsive to any contemporary treatment strategies [1]. Hence, there is a critical need for novel therapeutic targets and approaches for the treatment of heart failure. For a number of drug classes, clinical trials (reviewed in [2,3]) have demonstrated a positive effect on morbidity and mortality in patients with heart failure with reduced ejection fraction (HFrEF). However for patients with heart failure with preserved ejection fraction (HFpEF), to date, no therapy has been shown to improve outcomes [4–6].

In murine models of HF generated by transaortic constriction (TAC), data suggested that the inhibition of the enzyme phosphodiesterase type 5 (PDE5) reversed hypertrophy and improved EF [7–10]. However, these results have been controversial [11–14]. Still, based on the studies in the mouse [7–10], inhibition of PDE5 was proposed as a potential therapy for HFpEF. However, randomized controlled trials using the PDE5 inhibitor sildenafil in patients with HFpEF did not demonstrate any benefit compared to placebo [15,16].

The reason underlying the lack of beneficial effect of PDE5 inhibition for the treatment of HFpEF is unclear, but one potential is a lack of significant expression of the target protein in myocardial tissue. Indeed, there has been uncertainty regarding the expression of PDE5 in cardiomyocytes [10,11], and it has been proposed that inconsistent detection of PDE5 in the heart is due to variable selectivity of commercially available antibodies [11]. The present study examines PDE5 expression in tissue lysates from the left ventricle (LV) of two different mammalian models of HF, as well as humans with and without HF, using murine and bovine lung as a positive control, by one- and two-dimensional SDS-PAGE in a manner that is molecular weight and isoelectric form specific.

## Methods

### Ethics Statement

Investigations using the human ventricular samples conformed to the principles outlined in the Helsinki Declaration of the World Medical Association. The ethical review boards of the Mayo Clinic (IRB) and the University of Sydney (Human Research Ethics Committee (HREC); Sydney, Australia) approved procurement and handling of the human cardiac material.

For tissue from failing hearts, all subjects provided written informed consent using a consent form approved by the IRB of the Mayo Clinic IRB#06-005671.

Tissues for the healthy donor controls were provided by Dr Cris dos Remedios; human tissues were obtained with approval from the Human Research Ethics Committees of the University of Sydney (approval numbers #09-2009-12146 and #2012/2814). Written informed consent from the donor or the next of kin was obtained for use of these samples in research.

Animal experiments were performed in accordance with the American Physiological Society principles for ethical treatment of animals, using a protocol approved by the Mayo Clinic Institutional Animal Care and Use Committee.

### Mouse—Minimally Invasive TAC

Eight-week-old male C57/BL6 mice were subjected to minimally invasive TAC as previously described [17,18]. Sham mice underwent an identical procedure without placement of a suture. As previously described, TAC produces a variable phenotype with some mice developing a HF phenotype characterized by severe systolic dysfunction and pulmonary congestion and others developing a compensated phenotype with hypertrophy but without systolic dysfunction or pulmonary congestion [18,19]. We have previously demonstrated that an ejection fraction (EF) <65% at three weeks post-TAC reliably predicts the ultimate HF phenotype [18,19]. Thus, at three weeks post-TAC, mice underwent echocardiography and mice with established remodeling and systolic dysfunction (EF<65%) were classified as having HF (TAC-HF) while mice with preserved EF were classified as having compensated hypertrophy (TAC-COMP). The hemodynamic characteristics of the mice are displayed in S1 Table.

### Canine Model of HFpEF

HFpEF was modeled in elderly (≈8–13 years) mongrel dogs by induced hypertension (bilateral renal wrapping) and administration of an aldosterone agonist (desoxycorticosterone pivalate) to accentuate cardiac remodeling (old hypertensive, Old HTN). An aortic catheter was implanted for blood pressure measurement as previously described [20,21] and chronic hypertension was confirmed over a study duration of 8 weeks. Young normal dogs underwent sham surgery and aortic catheter placement. Compared to young normal dogs, Old HTN dogs displayed concentric LV hypertrophic remodeling, impaired LV relaxation and increased passive LV chamber stiffness (S2 Table), consistent with diastolic dysfunction [22,23].

### Tissue Collection

Tissue samples from all animals were weighed, flash-frozen in liquid nitrogen and stored at -80°C.

### Human Samples

Human left ventricular tissue samples were collected at the time of cardiac transplantation at the Mayo Clinic, in accordance with IRB#06-005671. Consent was obtained from all patients prior to sample collection. The obtained samples were rapidly frozen in liquid nitrogen and stored at -80°C until use. Human age matched controls were obtained from Dr Cris dos Remedios; tissues were with approval from the Human Research Ethics Committees of the University of Sydney (approval numbers #09-2009-12146 and #2012/2814). Written informed consent from the donor or the next of kin was obtained for use of these samples in research. Patient characteristics are listed in Table 1.

**Table 1:** Human Samples/Patient Characteristics.

*Sample*	*Age*	*Sex*	*EF*	*Cause of Death/Medications*
Control 1	52	M		Cerebral hemorrhage
Control 2	61	M		Unknown
Control 3	56	M		Cerebral hemorrhage
Control 4	55	M		Aortic aneurysm
Control 5	44	M		Unknown
Control 6	47	M		Unknown
Control 7	65	M		Aortic aneurysm
Control 8	54	M		Crush injury
HF 1	60	M	10	Aspirin, carvedilol, furosemide, simvastatin
HF 2	75	M	20	Amiodarone, carvedilol, digoxin, furosemide, metolazone, potassium, spironolactone
HF 3	68	M	35	Aspirin, furosemide, lisinopril, metoprolol, simvastatin
HF 4	39	M	20	Amiodarone, aspirin, carvedilol, furosemide, lisinopril, potassium, spironolactone
HF 5	74	M	25	Aspirin, carvedilol, digoxin, furosemide, isosorbide dinitrate, lisinopril, niacin
HF 6	55	M	15	Aspirin, carvedilol, digoxin, furosemide, lisinopril, potassium, spironolactone

### 1D and 2D SDS-PAGE

The techniques for both one- and two-dimensional (1-D, 2-D) immunoblotting have been previously published [18,24,25]. Briefly for both cardiac and lung samples, proteins for SDS-PAGE studies were extracted by homogenization on ice using 50% glycerol, 0.4M Bis-Tris pH7, 4% LDS, 2 mM EDTA, 0.075% Coomassie G250 and 0.25% Phenol Red (NuPAGE LDS Sample Buffer, Invitrogen, Carlsbad, CA). Prior to sample loading, a reducing agent, 10 mM DTT (NuPAGE sample reducing agent, Invitrogen, Carlsbad, CA) was added. For Western blotting, protein extracts were resolved by 29:1 10% SDS-PAGE and then transferred to a Hybond PVDF membrane (GE Healthcare, Piscataway, NJ). Membranes were probed with anti-PDE5 antibodies (Santa Cruz, Cell Signaling and a non-commercial rabbit polyclonal anti-PDE5 antibody provided by Joseph A Beavo [26] as well as an anti-PKG antibody (Cell Signaling).

2-D SDS-PAGE was used to probe mouse and human LV as well as lung homogenates for PDE5. Proteins for 2D SDS-PAGE were extracted by homogenization on ice using 7M urea, 2M thiourea, 4% CHAPS, 1% DTT, 2% 3–10 immobilized pH gradient buffer, protease and phosphatase inhibitors (Roche Diagnostics, Mannheim, Germany). Homogenates were first processed using a 2D-Clean-Up Kit prior to resolution in the first dimension (Amersham Biosciences, Piscataway, NJ). 3–11 NL IPG strips were rehydrated overnight with protein homogenate in acidic protein rehydration buffer (7M Urea, 2M Thiourea, 2% CHAPS, 0.5% 3.5–5.0 IPG buffer, 0.002% bromophenol blue and protease inhibitor). The isoelectric focusing was performed with an Ettan IPGphor 3 Isoelectric Focusing Unit. After isoelectric focusing, all gel strips were equilibrated in 6M urea, 50mM Bis-Tris, pH 6.4, 30% glycerol, 2% SDS, and 0.002% bromophenol blue containing first 10 mM dithiothreitol for 15 minutes and then 2.5% iodoacetamide for 15 minutes. The second dimension SDS-PAGE resolution was performed using a 29:1 10% polyacrylamide gel. Proteins were then transferred to Hybond PVDF membrane (GE Healthcare, Piscataway, NJ) and then probed with anti-PDE5 antibodies.

### qRT-PCR

RNA was extracted using the RNeasy extraction kit (Qiagen) according to the manufacturer’s protocol. The extracted RNA was quantified and then 1μg RNA was reverse transcribed to synthesize cDNA using the M-MLV Reverse Transcriptase Kit (Invitrogen) and random primers (Oligo d(T), Invitrogen). Then after initial denaturation with LightCycler 480 Probes Master mix (Roche), 50ng of the cDNA underwent 40 rounds of amplification (LightCycler 480, Roche) using primers for PDE5 and actin (500nM, Hs.PT.58.4779584 & Hs.PT.56a.1565177, Integrated DNA Technology). Data were analyzed and relative expression determined using the comparative cycle threshold (Ct) method (2^−ΔΔct^), and expression was normalized using ACTC1 as the housekeeping gene.

## Results

### PDE5 Expression in Murine Myocardium/Specificity of anti-PDE5 Antibodies

**Fig 1:**
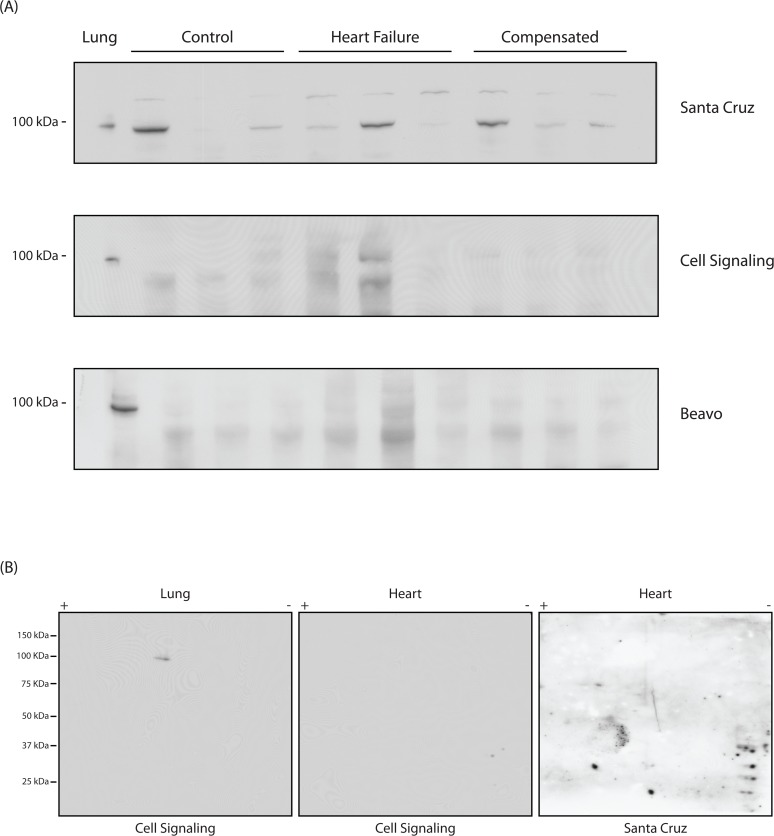
PDE5 is not detected in murine LV. (A) Western blots of LV samples from the murine demonstrate that PDE5 is detected in samples from the lung used as a positive control. For LV samples from normal (control), heart failure and compensated hypertrophy, there is non-specific binding of the anti-PDE5 antibody from Santa Cruz at a MW near the positive control, while PDE5 is not detected with the anti-PDE5 antibody from Cell Signaling or the rabbit polyclonal anti-PDE5 antibody provided by Joseph A Beavo, [26]. (B) Western blots of 2-D gels with the anti-PDE5 antibody (Cell Signaling) demonstrate that PDE5 is detected in the lung as 2 isoelectric variants, while PDE5 is not detected in cardiac tissue lysates (normal mice). However in cardiac tissue lysates, the anti-PDE5 antibody from Santa Cruz demonstrates non-specific binding.

To examine the specificity of the anti-PDE5 antibodies, we performed immunoblotting with cardiac LV cell lysates obtained from mouse heart (control, HF (TAC-HF), compensated hypertrophy (TAC-Comp)), and mouse lung (positive control) using the three different anti-PDE5 antibodies. In murine lung cell lysates, all antibodies recognized a single band at ∼98kDa, consistent with PDE5 (Fig. 1A). In cardiac muscle lysates, the Santa Cruz antibody was positive in some samples, at a number of different MWs, none of which matched the positive control (Fig. 1A). Although the anti-PDE5 antibody from Cell Signaling and the rabbit polyclonal anti-PDE5 antibody [26] recognized a single band in the lung, these antibodies did not identify any protein in cardiac muscle lysates (Fig. 1A). To further characterize antibody specificity, we immunoblotted two-dimensional gels (Fig. 1B). In lung tissue, immunoblotting with the Cell Signaling anti-PDE5 antibody identified 2 spots at a MW of ∼98kDa and a pI of ∼5.7 consistent with the predicted MW and pI of PDE5 as well as the presence of two isoelectric PDE5 variants, while PDE5 was not detected in cardiac muscle lysates. This is in contrast to the results obtained with the anti-PDE5 antibody obtained from Santa Cruz, which in cardiac muscle lysates identified a number of proteins, none of which were consistent with the predicted MW and pI of PDE5, which is similar to the 1-D immunoblot; from lane to lane, the MW and intensities of the bands detected with the Santa Cruz antibody were variable (Fig. 1A). These results suggest that the anti-PDE5 antibody obtained from Santa Cruz is poorly selective compared to the other anti-PDE5 antibodies, and thus, we used either the anti-PDE5 antibody obtained from Cell Signaling or the rabbit polyclonal anti-PDE5 antibody obtained from Joseph Beavo [26] for the remaining experiments.

### PDE5 Expression in Canine Myocardium

**Fig 2:**
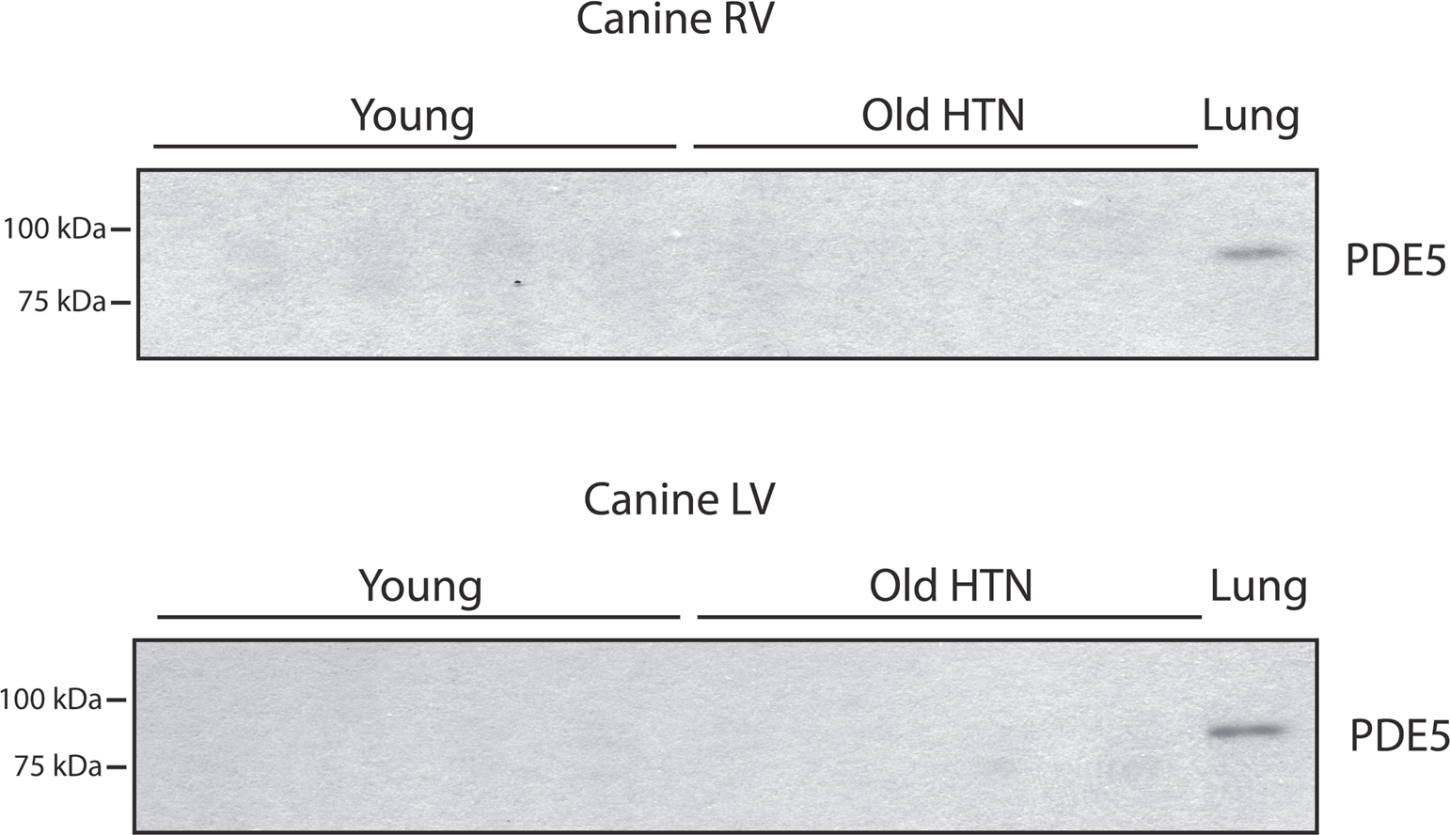
*PDE5 is not detected in cardiac LV and RV lysates from canines*. PDE5 was not detected in tissue lysates from the LV or RV in young normal (Normal) or old hypertensive canines (Old HTN) with diastolic dysfunction using the rabbit polyclonal anti-PDE5 antibody provided by Joseph A Beavo, [26]. Bovine lung is used as a positive control.

We next examined PDE5 expression in canine RV and LV homogenates. The old hypertensive (Old HTN) canines have been demonstrated to have diastolic dysfunction [22,23] and are a well-accepted and established large animal model of HFpEF. Similar to rodents, we were unable to detect PDE5 expression in myocardial tissue homogenates from either the RV or LV from young normal canines or the old hypertensive canines, while PDE5 was detected as a single band in the bovine lung sample used as a positive control (Fig. 2).

### PDE5 Expression in Human Myocardium

**Fig 3:**
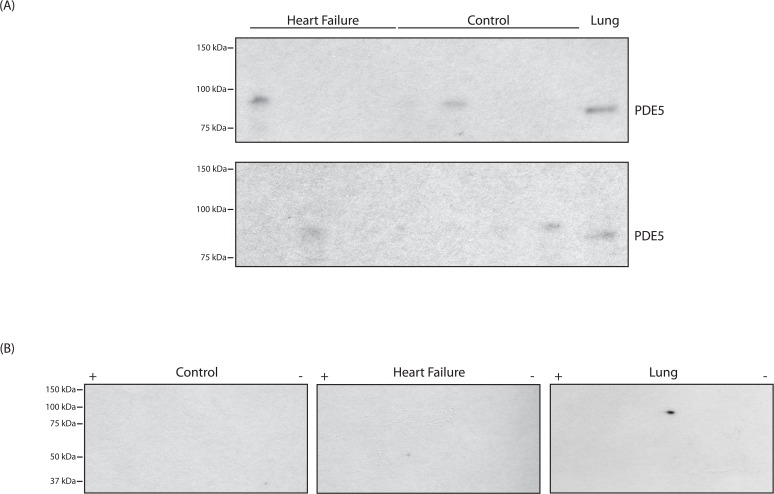
*PDE5 is not detected in LV samples from human controls or patients with heart failure*. (A) PDE5 is not detected by immunoblotting of human LV samples (heart failure (n = 6) and controls (n = 8); bovine lung is used as a positive control (rabbit polyclonal anti-PDE5 antibody provided by Joseph A Beavo, [26]). Upper blot—HF 1–3, Control 1–4 and lower blot—HF 4–6, Control 5-8 (refer to Table 1). (B) Western blots of 2-D gels with the anti-PDE5 antibody (Cell Signaling) demonstrate that PDE5 is detected in the bovine lung as 2 isoelectric variants, while PDE5 is not detected in cardiac tissue lysates of human LV samples (control (Control 2)) and heart failure (HF1)).

Finally, we examined PDE5 expression in humans with HF as well as normal controls. The clinical characteristics of the HF patients are displayed in Table 1, and controls were matched for both age and sex. Similar to our other results, we did not detect PDE5 expression in cardiac LV muscle lysates from either normal controls or humans with heart failure (Fig. 3A). Similar to our results from Western blots of 2-dimensional gels in the mouse, although PDE5 was detected as two isoelectric variants in the bovine lung, it was not detected in cardiac tissue lysates obtained from control or patients with HF (Fig. 3B).

We also examined PDE5 mRNA expression in the human LV tissue samples using qRT-PCR. In heart failure, compared to control, PDE5 expression was upregulated by 2.1±0.3-fold, in agreement with the results of others [27].

### PKG Expression

**Fig 4:**
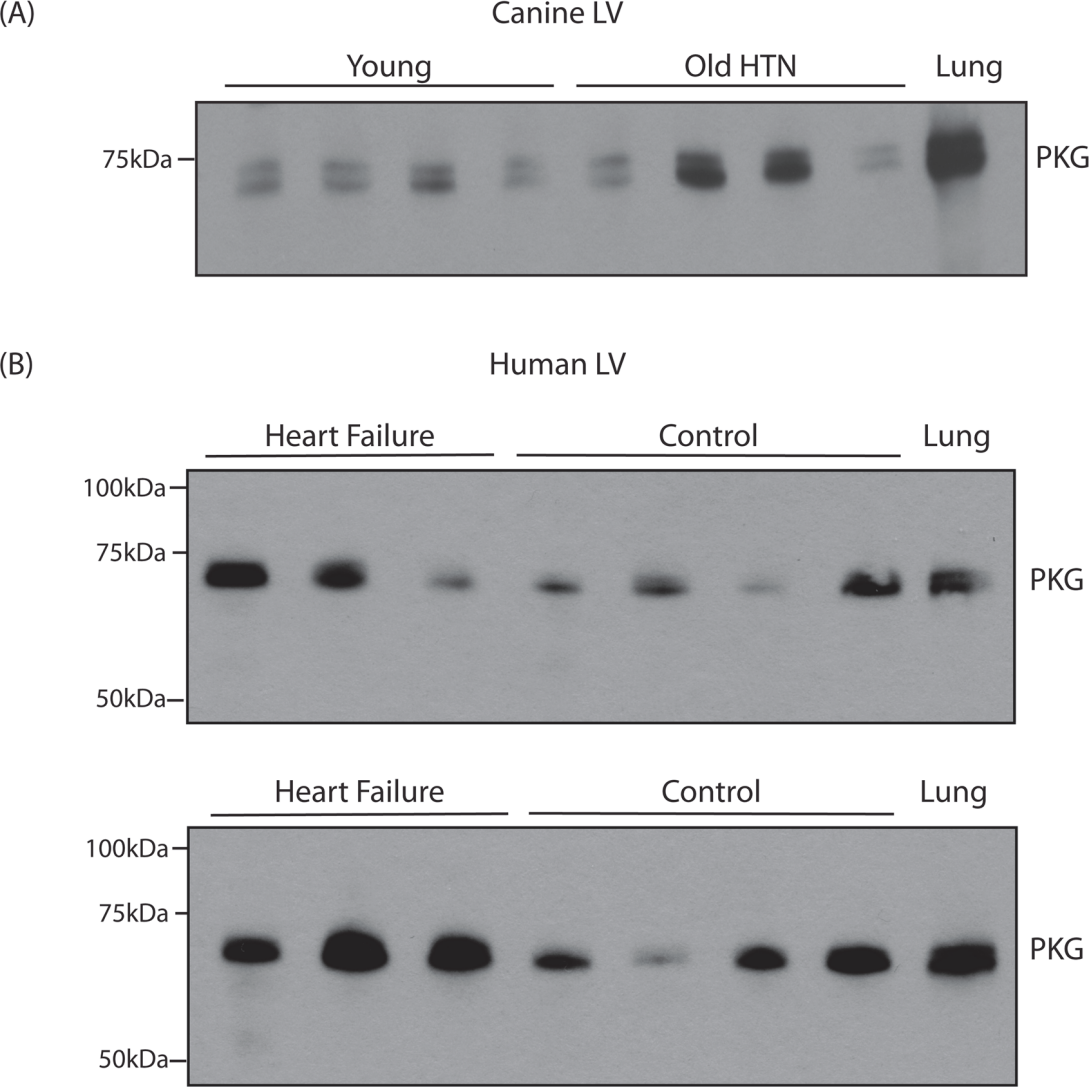
*PKG is detected in both canine and human LV samples*. (A) PKG is detected in LV tissue lysates in young normal (Normal), old hypertensive canines (Old HTN) with diastolic dysfunction, as well as bovine lung. (B) PKG is detected by immunoblotting of human LV samples (heart failure (n = 6) and controls (n = 8), as well as bovine lung. Upper blot—HF 1-3, Control 1-4 and lower blot—HF 4-6, Control 5-8 (refer to Table 1).

As described above, we were unable to detect the expression of the protein PDE5 in the cardiac tissue lysates (Figs. 1–3). One possible explanation is poor sample preparation. Although we would deem this unlikely, to rule out this possibility, we examined the expression of PKG. As expected and demonstrated in Fig. 4, PKG was detected in the lung as well as the LV samples.

## Discussion

The primary finding of our study is that we could not detect PDE5 in tissue homogenates from the LV of mice (normal, compensated hypertrophy or HF, Fig. 1), canines (LV and RV with and without HFpEF, Fig. 2) and human heart samples (normal controls or patients with HF, Fig. 3), while PDE5 was detected as a single band in lysates prepared from the murine and bovine lung used as a positive control. The NH_2_-terminal aa sequence of PDE5 is variable among species, but there is a high degree of identity for the remaining sequence [28]. For mouse and human PDE5, the first 20 (mouse) and 30 (human) aa of PDE5 are variable, but the remaining 841 aa are 96% identical, while the sequences of canine, bovine and human PDE5 are 97% identical. PDE5 is easily detected in the murine and bovine lung samples (Figs. 1–3). Thus, in cardiac tissue lysates of murine, canine and human, if PDE5 is present, there is no reason that anti-PDE5 polyclonal antibodies should not detect PDE5, other than expression being below the limit of detection.

Our data also demonstrate that in the normal murine and bovine lung, PDE5 is detected as two isoelectric variants. Isoelectric focusing and subsequent electrophoresis of tissue homogenates may assist in differentiating non-specific signals on immunoblotting by confirming presence of the expected isoelectic variants of PDE5 (Figs. 1B & 3B) and add a degree of specificity to our attempts at determining expression in different tissue and pathological states.

Others have reported that PDE5 is expressed in murine myocardium [7–10], and in addition, have demonstrated that sildenafil reduces cardiac hypertrophy in the TAC model of LV failure [8]. However, LV hypertrophy following TAC was not increased in the cardiac specific PKG KO mouse [11]. These investigators [11] demonstrated that PDE5 expression was not detected in murine cardiomyocytes, but PDE5 was detected in the lung and smooth muscle, which is similar to our results (Fig. 1) and those of others [12]. More recently using a mouse line that only expresses PKG in smooth muscle cells and cardiac myofibroblasts, investigators have demonstrated that inhibition of PDE5 with sildenafil did not decrease cardiac hypertrophy induced by the infusion of angiotensin II, but did significantly decrease fibrosis suggesting that inhibition of PDE5 in cells other than cardiac myocytes and smooth muscle was responsible for the antifibrotic effect [29]. These results are consistent with our findings and those of others, which demonstrate that PDE5 is not detected in cardiac tissue lysates [11,12].

It is known that in the myocardium, PDE5 is expressed in cell types other than cardiomyocytes [11,29]. Further, PDE5 mRNA is differentially expressed in heart vs. lung; in the whole heart, PDE5 mRNA expression is 100-fold less than in the lung, while in cardiomyocytes it is more than 1000-fold less than in the lung [9]. For lysates of the whole heart vs the lung, [PDE5] has been calculated to be ∼10nM vs. ∼250nM [30]. These investigators demonstrated that sildenafil did not affect cardiac contractility [31] and the small amount of PDE5 in the whole heart sample was derived from the PDE5 expressed in the vascular smooth muscle of the coronary arteries present in the whole heart lysates [30,31]. In the contractile myocardium, cardiomyocytes account for 90% of the cell mass and the remaining cell mass is predominantly fibroblasts [32]. We prepared our samples from small pieces of the muscular ventricular wall, and in these samples, the mass of cardiac myocytes far exceeds the mass of noncardiac cells [32], and therefore, protein expression in our cardiac samples would primarily reflect that of cardiomyocytes. Our results demonstrating that PDE5 was not detected in the murine LV samples are in agreement with those of others [11,30,31]. The lack of detectable PDE5 expression in the murine myocardium is also supported by data demonstrating that sildenafil does not influence cardiac contractility [29,31], and the inhibition of PDE5 with sildenafil does not increase LV cGMP content [8,9]. Additionally, without a positive control loaded on the same gel (refer to Fig. 1A), non-specific binding of the anti-PDE5 antibody could erroneously indicate that PDE5 is expressed in murine myocardium. Further although the effects of sildenafil are attributed to inhibition of PDE5, sildenafil inhibits other PDEs (PDE1 and PDE3) known to be expressed in the myocardium [13,14]; sildenafil inhibits recombinant PDE5, PDE1 and PDE3 with an IC_50_ of 6nM, 230nM and 30μm, respectively [13]. Thus, the antihypertrophic effect of sildenafil could be attributed to an inhibition of PDE1 or PDE3 combined with an antifibrotic effect on noncardiac and nonsmooth muscle cells within the heart [11,29].

Similar to our findings in the mouse, we could not detect PDE5 expression in tissue lysates from either the RV or LV from young normal or elderly hypertensive canines with HFpEF (Fig. 2). This contrasts with reports of others [33]; these investigators detected PDE5 in the LV of normal canines and demonstrated that PDE5 expression was reduced in tachycardia induced HF. In this study [33], a positive control (rat skeletal muscle) was provided, but the positive control was not loaded on the same gel as the samples from the canine LV. Further, multiple bands were present in the immunoblots of the canine LV samples, and in the control samples, PDE was defined as the band present at ∼98kDa.

Other investigators have reported that while PDE5 is not expressed in the myocardium of normal human RV and LV, hypertrophy in either chamber leads to upregulation of PDE5 expression [27,34,35]. These results are diametrically opposed to our findings (Fig. 3). Some of these studies have relied on immunohistochemistry, a method in which it is difficult to include a positive control in the same experiment. Further when PDE5 was examined with immunoblotting, both the intensity and MW of the reputed PDE5 signals were variable between lanes and no positive control was included, raising concerns that non-specific binding of the anti-PDE5 antibody (see Fig. 1) was attributed to PDE5 expression [34–36]. Additionally, Vandeput et al [13] demonstrated that sildenafil did not influence cGMP-hydrolytic activity in either normal or failing human myocardium, suggesting that the vast majority of cGMP-hydrolytic activity in human myocardium is attributable to PDE1 and PDE3.

Similar to others [27], we found that PDE5 mRNA is upregulated in heart failure. However, there are many instances in which changes in transcription are not reflected as a change in protein expression [37]. Since sildenafil inhibits the protein PDE5, our primary objective was to examine the protein PDE5, which was not detected in any of the cardiac tissue lysate examined. Although we cannot state whether the level of protein expression of PDE5 changed, our data clearly shows that for every condition examined, the level of PDE5 protein expression in our cardiac tissue lysates remained below the limit of detection using immunoblotting (Figs. 1–3).

In humans, the expression of PDE5 may be modulated during hypertrophy; i.e., below the limits of detection in the normal myocardium, upregulated during hypertrophy and then downregulated to below the limits of detection in end-stage heart failure. We were restricted to collecting tissue from humans at the time of transplant, and thus unable to assess PDE5 expression during compensated hypertrophy. In animal models, the prevailing dogma is that PDE5 expression in cardiomyocytes is abundant [7–10,33]. However, our data clearly show that we did not detect PDE5 expression in the LV samples of normal mice, mice with compensated hypertrophy or mice with heart failure (Fig. 1), as well as the RV and LV of young normal or the old hypertensive canines with diastolic dysfunction (Fig. 2), while PDE5 was detected in the lung samples used as a positive control and loaded on the same gel.

Using immunohistochemistry, Nagendran et al [27] demonstrated that in the normal human RV, PDE5 was not detected in the myocardium, and further, PDE5 was markedly upregulated in RV hypertrophy. These authors stated that there was insufficient tissue for immunoblotting in the majority of the patients, but an immunoblot is presented for a single control and single hypertrophied RV in the Supplemental data. Although in the normal RV, PDE5 was not detected with immunohistochemistry, PDE5 was detected on the immunoblot, and in RVH, the expression of PDE5 was markedly upregulated. However, this blot lacked a positive control. We cannot generalize our results in the LV to the RV. However in RVH, Nagendran et al [27] demonstrated PDE5 mRNA is upregulated by 15-fold. If in the heart, [PDE5] is ∼10nM [30] and during failure, is upregulated by 2-fold (our study), [PDE5] would be ∼20nM, which could be below the limits of detection using immunoblotting (Fig. 3). However if PDE5 is upregulated by 15-fold during hypertrophy [27], [PDE5] should be ∼180nM, which approaches the ∼250nM present in the lung. If this was the case, we should have detected PDE5 in mice with compensated hypertrophy (Fig. 1) as well as the old hypertensive canines (Fig. 2). However for every condition examined, our immunoblots demonstrate that in our LV samples, PDE5 is below the level of detection (Figs. 1–3). In addition, Corbin et al [30] calculated that the ratio of PKG/PDE5 is 6 in the whole heart vs 0.13 in the lung, and demonstrated that the ratio of expression of PKG in the heart/lung is ∼3. These data are consistent with our findings; in lung samples, we could easily detect PKG and PDE5, while in both human and canine LV samples, although the Western blot for PKG is positive (Fig. 4), there was no detectable expression of PDE5 (Figs. 2–3).

We believe accurate identification of tissue expression of the protein(s) targeted by pharmacologic interventions should guide our attempts at designing novel treatment strategies. In lysates prepared from the whole heart, a small amount of PDE5 can be detected due to PDE5 expression in the vascular smooth muscle [30,31], and in the myocardium, cardiomyocytes account for 90% of the cell mass [32]. However, the treatment of heart failure with inhibitors of PDE5 [15] is not based on PDE5 expression in the vasculature, but rather on the abundant expression of PDE5 in cardiomyocytes, and further, the beneficial effects on hypertrophy and heart failure are said to be due to the inhibition of PDE5 in cardiomyocytes [7–10,33]. For HFpEF, the RELAX trial [15] did not demonstrate a benefit of PDE5 inhibition with sildenafil, and similar results were reported for patients with a normal ejection fraction following an uncomplicated myocardial infarction [16]. The lack of detectable PDE5 expression in the human LV (Fig. 3) may explain the lack of benefit of PDE5 inhibition with sildenafil in these two clinical trials [15,16]. In the only clinical trial in which sildenafil was shown to be of benefit in HFpEF [38], a defining characteristic of the study population was marked pulmonary arterial hypertension, out of proportion to that observed in the more recent trials [15,16]. For the HFpEF patients in this trial [38], the pulmonary artery systolic pressure (PASP) was ∼37 mmHg, which in the sildenafil treated group, fell to ∼28 mmHg. There is no question that there is abundant PDE5 expression in the pulmonary vasculature [28,30,39], and for patients with pulmonary hypertension, inhibition of PDE5 will decrease PASP [39–42]. This raises the possibility that the improvements in the patients with both elevated PASP and HFpEF [38] were due to the inhibition of PDE5 in the pulmonary vasculature and resulting decrease in PASP. The trial published by Giannetta et al [43] randomized diabetic patients with no ischemia and normal LV function to sildenafil vs placebo. This study’s primary endpoint was LV torsional angle; sildenafil improved both torsion and strain. After 3 months of therapy (placebo vs sildenafil), there was no difference in LV mass and the end diastolic volume (EDV) indexes were identical; thus, the improvement in the calculated LV mass/volume could have been due to the differences in baseline patient characteristics rather than an effect of sildenafil. The RELAX trial [15] enrolled significantly more patients than the other trials that evaluated sildenafil as a therapy for HF [16,38,43]. Whether the beneficial effects of sildenafil in two of the smaller trials [38,43] were due to the smaller patient cohorts or the clinical characteristics of the patient populations is unclear. Nonetheless, our results demonstrate that the expression of PDE5 is below the limit of detection using immunoblotting in the LV of normal humans and humans with end-stage heart failure and would predict that for patients with heart failure, sildenafil therapy would have no clinical benefit.

Recently, Patrucco et al [29] demonstrated that in angiotensin II induced cardiac hypertrophy, sildenafil did not decrease either LV mass or cardiomyocyte size, or improve LV function. Sildenafil did significantly decrease cardiac fibrosis. However, the decrease in fibrosis was not due to inhibition of PDE5 in cardiomyocytes or smooth muscle cells. The identity of this other cell type that expresses PDE5 and is responsible for cardiac fibrosis is unknown. These results suggest that in humans with cardiac disease, sildenafil therapy could decrease LV fibrosis, but this hypothesis requires rigorous investigation.

### Summary

Our data clearly demonstrate that we are unable to detect PDE5 in any of the cardiac tissue lysates examined from humans or experimental models of HF, whereas PDE5 is present in the murine and bovine lung samples used as a positive control. These results indicate that if PDE5 is expressed in cardiac tissue, it is present in very low quantities [11,12]. Therefore in cardiac muscle, it is difficult to envision a role for PDE5 in the regulation of the cGMP-PKG signaling, and it is unlikely that PDE5 would be a suitable drug target for the treatment of cardiac hypertrophy. These results help explain the lack of a therapeutic effect in recent clinical trials examining the inhibition of PDE5 in patients with heart failure [15,16], which highlights the importance of the rigorous demonstration of the protein targeted by therapy in the normal and diseased heart prior to clinical trial design.

## Supporting Information

S1 Table Hemodynamics in Mice. Following TAC, compared to the sham group (Normal), the compensated group demonstrated cardiac hypertrophy (increase in the LV/BW), but EF remained above 60% and there was no significant increase in the lung/BW consistent with LV hypertrophy without HF, while the heart failure (HF) group demonstrated cardiac hypertrophy (increase in LV/BW), pulmonary congestion (increase in lung/BW) and a significant reduction in EF, consistent with a HF phenotype [18,19]. Data are mean±SEM; *, p<0.05 vs normal. BW (body weight), LV (left ventricle).
(DOCX)

S2 Table Hemodynamics in Dogs. Compared to young canines, old hypertensive (Old HTN) canines demonstrate a significant increase in LV mass, as well as both Ees (end systolic elastance) and β (diastolic stiffness coefficient), consistent with diastolic dysfunction and HFpEF [22,23]. Data are mean±SEM; *, p<0.05 vs Young.
(DOCX)
