# Supplementary material for: The Emperor's New Clothes: PDE5 and the Heart
Source: PLoS One. 2015 Mar 6;10(3):e0118664. doi: 10.1371/journal.pone.0118664 (PMC4351884; doi:10.1371/journal.pone.0118664)
Supplement: S1 Table — Following TAC, compared to the sham group (Normal), the compensated group demonstrated cardiac hypertrophy (increase in the LV/BW), but EF remained above 60% and there was no significant increase in the lung/BW consistent with LV hypertrophy without HF, while the heart failure (HF) group demonstrated cardiac hypertrophy (increase in LV/BW), pulmonary congestion (increase in lung/BW) and a significant reduction in EF, consistent with a HF phenotype [18,19]. Data are mean±SEM; *, p<0.05 vs normal. BW (body weight), LV (left ventricle). (DOCX) [file pone.0118664.s001.docx]

Supporting Table I. Hemodynamics in Mice

|  | Normal | Compensated | Heart Failure |
| --- | --- | --- | --- |
|  | (n=14) | (n=12) | (n=13) |
| EF (%) | 80±1 | 64±4* | 30±5* |
| LV/BW (mg/g) | 4.7±0.2 | 7.4±0.5* | 12.3±0.9* |
| Lung/BW (mg/g) | 5.7±0.2 | 6.3±0.4 | 16.5±1.6* |

Following TAC, compared to the sham group (Normal), the compensated group demonstrated cardiac hypertrophy (increase in the LV/BW), but EF remained above 60% and there was no significant increase in the lung/BW consistent with LV hypertrophy without HF, while the heart failure (HF) group demonstrated cardiac hypertrophy (increase in LV/BW), pulmonary congestion (increase in lung/BW) and a significant reduction in EF, consistent with a HF phenotype [18,19]. Data are mean±SEM; *, p<0.05 vs normal. BW (body weight), LV (left ventricle).
