# Supplementary material for: The Emperor's New Clothes: PDE5 and the Heart
Source: PLoS One. 2015 Mar 6;10(3):e0118664. doi: 10.1371/journal.pone.0118664 (PMC4351884; doi:10.1371/journal.pone.0118664)
Supplement: S2 Table — Compared to young canines, old hypertensive (Old HTN) canines demonstrate a significant increase in LV mass, as well as both Ees (end systolic elastance) and β (diastolic stiffness coefficient), consistent with diastolic dysfunction and HFpEF [22,23]. Data are mean±SEM; *, p<0.05 vs Young. (DOCX) [file pone.0118664.s002.docx]

Supporting Table II. Hemodynamics in Dogs

|  | Young | Old HTN |
| --- | --- | --- |
|  | (n=13) | (n=9) |
| EF (%) | 55±2 | 54±5 |
| LV Mass (g) | 86±2 | 105±8* |
| Ees (mm Hg/ml) | 1.06±0.06 | 3.2±0.3* |
| β (mm Hg/ml) | 0.016±0.002 | 0.044±0.010* |

Compared to young canines, old hypertensive (Old HTN) canines demonstrate a significant increase in LV mass, as well as both Ees (end systolic elastance) and β (diastolic stiffness coefficient), consistent with diastolic dysfunction and HFpEF [22,23]. Data are mean±SEM; *, p<0.05 vs Young.
